# Comparison of the risk of hospitalisation among BA.1 and BA.2 COVID‐19 cases treated with sotrovimab in the community in England

**DOI:** 10.1111/irv.13150

**Published:** 2023-05-28

**Authors:** Katie Harman, Sophie Grace Nash, Harriet H. Webster, Natalie Groves, Jo Hardstaff, Jessica Bridgen, Paula B. Blomquist, Russell Hope, Efejiro Ashano, Richard Myers, Sakib Rokadiya, Susan Hopkins, Colin S. Brown, Meera Chand, Gavin Dabrera, Simon Thelwall

**Affiliations:** ^1^ UKHSA COVID‐19 National Epidemiology Cell London UK; ^2^ UKHSA Genomics and Public Health Analysis London UK; ^3^ UKHSA Outbreak Surveillance Team London UK; ^4^ UKHSA HCAI, Fungal, AMR, AMU & Sepsis Division London UK; ^5^ UKHSA COVID‐19 Therapeutics London UK; ^6^ UKHSA Chief Medical Advisor London UK; ^7^ UKHSA HCAI, Fungal, AMR, AMU & Sepsis Division & NIHR Health Protection Research Unit in Healthcare Associated Infections and Antimicrobial Resistance London UK

**Keywords:** COVID‐19 therapeutics, neutralising monoclonal antibodies, SARS‐CoV‐2, severity

## Abstract

There are concerns that sotrovimab has reduced efficacy at reducing hospitalisation risk against the BA.2 sub‐lineage of the Omicron SARS‐CoV‐2 variant. We performed a retrospective cohort (n = 8850) study of individuals treated with sotrovimab in the community, with the objective of assessing whether there were any differences in risk of hospitalisation of BA.2 cases compared with BA.1. We estimated that the hazard ratio of hospital admission with a length of stay of 2 days or more was 1.17 for BA.2 compared with BA.1 (95%CI 0.74–1.86). These results suggest that the risk of hospital admission was similar between the two sub‐lineages.

## 1 INTRODUCTION

The use of therapeutic agents for the treatment of COVID‐19 is a rapidly evolving field of research where several new and existing treatments have been licensed to help treat COVID‐19 infection in people who are at the highest risk of developing severe outcomes. In the United Kingdom (UK), neutralising monoclonal antibody (nMAb) sotrovimab was approved in December 2021 for use in symptomatic people who do not require oxygen but are at increased risk of severe infection.^1^ It was licensed to treat SARS‐CoV‐2 infection both for the people in the community and those hospitalised, aged 12 years and older who are at high risk of developing severe COVID‐19 disease.^2^

Initial analyses of sotrovimab have shown that its administration significantly reduced the risk of hospitalisation or death in people with mild to moderate infection who had at least one risk factor for developing severe outcomes.^3^, ^4^ However, findings from in vitro studies have raised concerns that sotrovimab may have reduced clinical efficacy for people with the BA.2 sub‐lineage of the Omicron (B.1.1.529) variant because of mutations in the spike protein causing reduced neutralising ability.^5^, ^6^ These concerns led to the withdrawal of sotrovimab authorisation in several countries including the United States of America.^7^ Sotrovimab was licensed in the UK as the third‐line recommended treatment for people in the hospital^8^ and the first‐line treatment for people in the community until November 2022.^9^ An initial analysis by the UK Health Security Agency (UKHSA) found that there was no increased risk of attendance or admission to hospital; however, this was limited by reduced follow up time.^10^ Therefore, this study sought to assess whether there were any differences in hospital admission among people who were treated with sotrovimab in community settings with BA.2 compared with those with BA.1 sub‐lineages.

## 2 METHODS

Information about individuals treated with sotrovimab in the community, excluding those treated after testing positive as a hospital inpatient, as submitted by the National Health Services (NHS) Trusts up to 24 May 2022, were extracted from Blueteq software and linked deterministically by an NHS number to the laboratory confirmed COVID‐19 cases in England from UKHSA's Second Generation Surveillance System (SGSS). This incorporates data on variant status from genotyping, S‐gene target data and whole genome sequencing (WGS).^11^ Vaccination status of these cases was then obtained by linking to the National Immunisation Management Service (NIMS) and categorised based on the receipt of vaccine at least 2 weeks prior to the positive specimen date. Cases were then linked to the NHS Digital's Emergency Care Data Set (ECDS) and Secondary Uses Service (SUS) for information about their hospital admissions as described elsewhere.^12^

Genotyping PCR can identify the Omicron variant but is unable to distinguish sub‐lineages. However, the S‐gene target status can be used in conjunction with genotyping PCR results to identify BA.1 and BA.2 for samples tested with the TaqPath assay, with BA.1 being associated with S‐gene target failure (SGTF) and BA.2 with S‐gene target positivity (SGTP) or used solely when Omicron was the dominant variant in circulation.^12^ The study therefore included: (1) all sequencing‐confirmed BA.1 and BA.2 cases; (2) between 1 January and 24 January 2022 when Delta (B.1.617.2) variant was prevalent, SGTF/SGTP information was only used if genotyping PCR results indicated Omicron; and (3) from 25 January to 3 May 2022 all SGTF/SGTP cases were included irrespective of genotyping PCR status as predictive values of SGTF/SGTP to call BA.1/BA.2 were ≥95%.^13^

To assess the relative severity of those infected with BA.2 variant SARS‐CoV‐2 compared with BA.1 variant, we used stratified Cox regression to calculate hazard ratios (HR) of the hospitalisation between 1–14 days after treatment date. Hospital attendances beginning on the day of treatment were excluded to ensure that attending for treatment, administered as a single dose, was not captured. Cox regression was chosen to account for censoring at 14 days after the date of treatment. Hospital admission was defined as: (1) hospital admission with either a length of stay of 2 days or more, or the person died in hospital, (2) hospital admission with either a length of stay of 2 days or more or the person died in hospital and the mention of COVID‐19 ICD‐10 codes (U071/U072), (3) hospital admission of any length of stay, or the person died in hospital. All definitions were excluding admissions for injury‐related reasons as identified by the primary complaint code on an emergency care record. Age and vaccination status were considered to be priori confounders and were thus included in the regression model. Age was adjusted for by including an age group and a linear term to account for residual confounding in age groups.^14^ A number of difficult‐to‐quantify time‐varying factors were considered likely to affect the probability of being admitted to a hospital at different time points. These included, but were not limited to, the level of bed‐occupancy in a given hospital, the number of healthcare workers available and social distancing behaviours of clinically vulnerable people. To account for this, the models were stratified by specimen week, allowing separate baseline hazard functions per week. All analyses were performed in R (version 4.1.2, R Foundation for Statistical Computing, Vienna, Austria).

## 3 RESULTS

Between 01 January and 26 April 2022, 20,274 records for community prescription of sotrovimab were received, 19,149 (94.5%) of which was successfully linked to the COVID‐19 case episodes list. A total of 8850 (46.2%) had BA.1 or BA.2 variant classification data available and were therefore included in the analysis, 4285 and 4565 for BA.1 and BA.2 respectively (Table 1).

**TABLE 1:** Demographic characteristics of COVID‐19 cases treated with sotrovimab in the community with Omicron lineage BA.1 or BA.2. England, 1 January to 26 April 2022 (n = 8850^a^).

Demographic		BA.1	BA.2	*p* value^c^
Variant classification	Validated whole genome sequencing	3230 (75.4%)	3566 (78.1%)	<0.05
	Genotyping + S‐Gene information	797 (18.6%)	698 (15.3%)	
	S‐Gene information only	258 (6.0%)	301 (6.6%)	
Sex	Female	2587 (60.4%)	2725 (59.7%)	0.53
	Male	1698 (39.6%)	1840 (40.3%)	
Age (years)	10–19	77 (1.8%)	62 (1.4%)	<0.05
	20–29	232 (5.4%)	190 (4.2%)	
	30–39	602 (14%)	465 (10.2%)	
	40–49	879 (20.5%)	735 (16.1%)	
	50–59	981 (22.9%)	986 (21.6%)	
	60–69	815 (19%)	1013 (22.2%)	
	70–79	556 (13%)	849 (18.6%)	
	≥80	143 (3.3%)	265 (5.8%)	
Ethnic group	Asian	247 (5.8%)	194 (4.2%)	0.01
	Black	101 (2.4%)	99 (2.2%)	
	Mixed/Other	84 (2.0%)	85 (1.9%)	
	Unknown	103 (2.4%)	96 (2.1%)	
	White	3750 (87.5%)	4091 (89.6%)	
Region	East Midlands	485 (11.3%)	441 (9.7%)	<0.05
	East of England	752 (17.5%)	869 (19.0%)	
	London	626 (14.6%)	787 (17.2%)	
	North East	245 (5.7%)	261 (5.7%)	
	North West	538 (12.6%)	489 (10.7%)	
	South East	593 (13.8%)	661 (14.5%)	
	South West	308 (7.2%)	357 (7.8%)	
	West Midlands	456 (10.6%)	449 (0.098%)	
	Yorkshire and Humber	282 (6.6%)	251 (5.5%)	
Indices of multiple deprivation (IMD) quintile	1 (Most deprived)	590 (13.8%)	536 (11.7%)	<0.05
	2	736 (17.2%)	778 (17.0%)	
	3	909 (21.2%)	993 (21.8%)	
	4	996 (23.2%)	1057 (23.2%)	
	5 (Least deprived)	1054 (24.6%)	1201 (26.3%)	
Vaccination status^b^	Unvaccinated or <28 days after first dose	62 (1.4%)	56 (1.2%)	0.31
	≥28 days after first dose	44 (1.0%)	32 (0.7%)	
	≥14 days after second dose	4136 (96.5%)	4432 (97.1%)	
	≥14 days after third dose	43 (1.0%)	45 (1.0%)	

^a^ Cases in study period excluded from study and not shown in table: 1125 sotrovimab records failed to match on NHS number to COVID‐19 cases data, 14 missing data on key covariates, 212 with incomplete follow‐up time.

^b^ Vaccination status was categorised based on receipt of vaccine at least 2 weeks prior to positive specimen date.

^c^ Chi‐squared test for independence.

The patient characteristics were broadly similar between the two cohorts, with the same proportions by sex. The BA.1 cohort had a younger age distribution compared with the BA.2, where the median age of individuals with BA.1 was 53 years (interquartile range 41–64) compared with 58 years for BA.2 (interquartile range 46–69). The geographical breakdown was broadly similar between the two groups; however, slightly more of the BA.2 cohort resided in London compared with the BA.1 cohort (p < 0.05); whereas, a slightly larger proportion of the BA.1 cohort resided in more‐deprived areas compared with the BA.2 (*p* < 0.05). Admitted patients were in older age groups for both variants, and the majority were ≥14 days after their second vaccination dose (Table 2).

**TABLE 2:** Hospitalisation^a^ by age and vaccination status for COVID‐19 cases treated with sotrovimab, with the Omicron lineage BA.1 or BA.2 (n = 8850).

Characteristic		BA.1			BA.2		
		Hospitalised	Not hospitalised	*p* value^c^	Hospitalised	Not hospitalised	*p* value^c^
Age	10–19	0 (0.0%)	77 (100.0%)	<0.05	1 (1.6%)	61 (98.4%)	0.13
	20–29	1 (0.4%)	231 (99.6%)		4 (2.1%)	186 (97.9%)	
	30–39	8 (1.3%)	594 (98.7%)		2 (0.4%)	463 (99.6%)	
	40–49	10 (1.1%)	869 (98.9%)		10 (1.4%)	725 (98.6%)	
	50–59	18 (1.8%)	963 (98.2%)		16 (1.6%)	970 (98.4%)	
	60–69	22 (2.7%)	793 (97.3%)		15 (1.5%)	998 (98.5%)	
	70–79	25 (4.5%)	531 (95.5%)		21 (2.5%)	828 (97.5%)	
	≥80	7 (4.9%)	136 (95.1%)		8 (3%)	257 (97.0%)	
Vaccination status^b^	Unvaccinated or <28 days after first dose	2 (3.2%)	60 (96.8%)	0.16	3 (5.4%)	53 (94.6%)	0.12
	≥28 days after first dose	3 (6.8%)	41 (93.2%)		0 (0.0%)	32 (100.0%)	
	≥14 days after second dose	85 (2.1%)	4051 (97.9%)		74 (1.7%)	4358 (98.3%)	
	≥14 days after third dose	1 (2.3%)	42 (97.7%)		0 (0.0%)	45 (100.0%)	

^a^ Hospitalisation according to outcome definition 1, where cases are individuals admitted ≥2 days or died in hospital.

^b^ Vaccination status was categorised based on receipt of vaccine at least 2 weeks prior to positive specimen date.

^c^ Chi‐squared test for independence.

The risk of hospital admission with a length of stay of 2 days or more, 1–14 days after the community treatment with sotrovimab, showed no statistically significant difference between BA.2 and BA.1 sub‐lineages (HR = 1.17, 95% CI 0.74–1.86; Table 3). Sensitivity analyses restricting to hospital records that stated COVID‐19 ICD‐10 codes on admission for greater specificity also showed no statistically significant difference in hospitalisation risk in BA.2 cases compared with BA.1 (HR = 0.98, 95% CI 0.58–1.65). Furthermore, admissions with any length of stay also did not substantially alter the estimate of the relative risks of admission (HR = 1.02, 95% CI 0.70–1.47).

**TABLE 3:** Risk of hospitalisation for COVID‐19 cases treated with sotrovimab in the community with Omicron lineage BA.1 or BA.2. England, 1 January to 26 April 2022.

Outcome	Count of outcome in BA.1 cases	Count of outcome in BA.2 cases	Adjusted hazard ratio (95% CI)^a^
Hospital admission with length of stay ≥2 days	91	77	1.17 (0.74–1.86)
Hospital admission with length of stay ≥2 days and mention of COVID‐19 ICD‐10 codes	73	62	0.98 (0.58–1.65)
Hospital admission of any length of stay	133	140	1.02 (0.70–1.47)

^a^ Adjusted hazard ratios and 95% confidence intervals (CI) from Cox regression models comparing the risk of admission of cases with BA.2 compared with BA.1. The models were stratified for specimen week, and additionally using regression adjustments for age group, linear effect in age and vaccination status, to account for confounders.

## 4 DISCUSSION

These results suggest that there is no statistically significant difference in the risk of hospital admission between BA.1 and BA.2 cases treated with sotrovimab in this English community cohort. This absence of difference persists when restricting to those admitted with a diagnosis of COVID‐19.

These findings are supported by the access to high quality data on therapeutics prescribed to COVID‐19 cases linked to national datasets, permitting a timely nationwide cohort study. Ascertaining case variant status through multiple methods allowed for a greater sample size than would otherwise be possible. Stratifying by specimen week and removing cases with insufficient follow‐up time aimed to mitigate the reporting lags observed in routine hospitalisation surveillance data, but some bias may remain.

There are several limitations to this study; primarily, NHS numbers were required to link the therapeutic data to the COVID‐19 cases data, and subsequent vaccination and hospitalisation data, which meant 1125 community sotrovimab records, were excluded from the analysis. This analysis was also limited by the overall number of cases available for inclusion, with variable testing guidance in England throughout the period and restrictions in free community testing from 1 April 2022. Whilst therapeutic agent recipients and hospitalised cases are prioritised for both testing and sequencing,^15^ the period saw a significant reduction in sequencing capacity, which may have limited the study size. Additional limitations include the lack of information on co‐morbidities, the date of onset of the symptoms for cases, and alternative measures of clinical severity such as people requiring ventilator support or admittance to intensive care units. Furthermore, the vaccination data available did not include the spring booster doses at the time of analysis.

It remains possible that sotrovimab has lower clinical effectiveness for people with both BA.1 and BA.2 compared with previously circulating variants such as Delta (B.1.617.2); however, because of the lack of cases of BA.2 and Delta occurring in the same time period, it is not possible to compare the severe outcomes following sotrovimab treatment between these cases.^13^

Although this analysis shows that there is no apparent reduction in sotrovimab effectiveness at reducing the risk of hospitalisation against BA.2 compared with BA.1 in this cohort, it is important that sotrovimab continues to be monitored so that treatment guidance can be updated when necessary. This is particularly important as new variants emerge, such as the BA.4 and BA.5 lineages of Omicron.

## AUTHOR CONTRIBUTIONS

Katie Harman, Simon Thelwall and Gavin Dabrera conceived and designed the study. Katie Harman, Harriet H Webster, Natalie Groves, Jo Hardstaff, Jessica Bridgen, Paula B. Blomquist, Russell Hope, Efejiro Ashano, Richard Myers, Sakib Rokadiya, Susan Hopkins, Colin S Brown, Meera Chand were involved in data curation and resources. Katie Harman prepared the final dataset and performed the analysis, supported by Sophie Grace Nash and Harriet H Webster. Katie Harman, Sophie Grace Nash and Simon Thelwall drafted the first version of the manuscript. All authors read, revised and approved the final version of the manuscript.

## CONFLICT OF INTEREST STATEMENT

GD declares that his employer's predecessor organisation, Public Health England, received funding from GlaxoSmithKline for a research project related to influenza antiviral treatment. This preceded and had no relation to COVID‐19, and GD had no role in and received no funding from the project. All other authors report no potential conflicts.

## ETHICS STATEMENT

UKHSA has legal permission, provided by the Regulation 3 of The Health Service (Control of Patient Information) Regulations 2002 to process confidential patient information under Section 3 sub‐sections (1)(a–c), (1)(d)(i and ii) and (3) as part of its outbreak response activities. This study falls within the research activities approved by the UKHSA Research Ethics and Governance Group.

### PEER REVIEW

The peer review history for this article is available at https://www.webofscience.com/api/gateway/wos/peer-review/10.1111/irv.13150.

## Data Availability

The individual‐level nature of the data used risks individuals being identified or being able to self‐identify if the data are released publicly. Requests for access to these non‐publicly available data should be directed to UKHSA.
